# Can We Protect Those We Care for in A Pandemic? - Prevalence of Neutralizing Antibodies against SARS-CoV-2 in Nursing Homes

**DOI:** 10.14336/AD.2021.0217

**Published:** 2021-06-01

**Authors:** Harald Willschke, Thomas Wochele-Thoma, Atanas G. Atanasov, Elisabeth Klager, Christian Haslinger, Maria Kletecka-Pulker, Daniel Laxar, Care Ay, Thomas Öhlinger, Oliver Kimberger, Adi Steinrigl, Barbara Holzer, Florian Heger, Alexander Indra

**Affiliations:** ^1^Ludwig Boltzmann Institute of Digital Health and Patient Safety, Vienna, Austria.; ^2^Department of Anaesthesia and General Intensive Care, Medical University Vienna, Austria.; ^3^Caritas Erzdiözese Wien, Vienna, Austria.; ^4^Institute of Genetics and Animal Biotechnology of the Polish Academy of Sciences, Jastrzebiec, Magdalenka, Poland.; ^5^Institute of Neurobiology, Bulgarian Academy of Sciences, Sofia, Bulgaria.; ^6^Department of Pharmacognosy, University of Vienna, Vienna, Austria.; ^7^Hygienefachkraft-unlimited, Vienna, Austria.; ^8^AGES - Österreichische Agentur für Gesundheit und Ernährungssicherheit GmbH, Institute for Veterinary Disease Control, Mödling, Austria.; ^9^AGES - Österreichische Agentur für Gesundheit und Ernährungssicherheit GmbH, Institute for medical Microbiology and Hygiene Vienna, Vienna, Austria.; ^10^Paracelsus Medical University of Salzburg, Salzburg, Austria.

**Keywords:** SARS-CoV-2, SARS-CoV-2 neutralizing antibodies, SARS-CoV-2 tests, COVID 19, nursing homes

## Abstract

In December 2019, the People's Republic of China and the World Health Organization first reported on a cluster of pneumonia with an unknown cause. Nine months later more than 1.4 million people have died from COVID 19. In this work, the effects of the COVID 19 pandemic on five nursing homes in Austria, which cared for 889 residents in the first half of 2020, were examined. The research question was whether the measures taken were appropriate to prevent an outbreak within the individual facilities. To detect previously unrecognized infections, the present study evaluated the prevalence of neutralizing antibodies against the SARS-CoV-2 virus in residents and employees of the nursing homes. Following the analysis of blood samples, the prospectively collected data was connected to data from screening examinations and data from contact tracing. The present study demonstrated an overall prevalence of neutralizing antibodies against the SARS-CoV-2 virus in nursing homes of 3.7%. Whereas the prevalence in those facilities that have never been hit by an outbreak is 0%, the prevalence in those facilities with an outbreak is up to 4.9%. Neutralizing antibodies against SARS-CoV-2 were detected in 35 persons. A retrospective analysis of all 5 included nursing homes demonstrated that upon regular clinical screening in combination with PCRs an infection with SARS-COV-2 was detected in 66 residents and 24 employees from different professional groups. In only 25 of the 35 persons with neutralizing antibodies against SARS-CoV-2 an infection was proven in advance. This study suggests that specific measures can prevent transmission within a health care facility. Nevertheless, the results also show that a risk reduction to 0% cannot be achieved. In preparation for further pandemic waves there is still the need to reduce the probability of a transmission in nursing homes with specific test strategies.

Human history is rich in descriptions of infectious diseases, epidemics, and occasionally pandemics. Bubonic plague, cholera, typhus, yellow fever and smallpox have led to devastating infectious diseases spreads time and again since ancient times. Europe, for example, has been hit several times by outbreaks of *Yersinia pestis* infections [1]. The oldest account of the plague can be found in the Bible in the second book of Samuel (Chapeter 25, Verse 15, old Testament): "So the Lord sent a plague upon Israel from that morning until the appointed time, and seventy thousands of the people from Dan to Beersheba died”. In contrast to the first pandemic wave, the other great waves of the plague in the Middle Ages are well documented by many authors [2]. While in many European cities the dead of the plague are still commemorated by monuments, one looks in vain for monuments that are supposed to commemorate what is probably the most devastating infectious disease of all time. Between 1918 and 1920 up to 100 million people worldwide died of the Spanish flu, an infection with an H1N1 influenza virus. 100 years later, the world is again in the middle of a pandemic [3-7]. On December 31, 2019, the People's Republic of China and the World Health Organization reported on a cluster of pneumonia with an unknown cause in the metropolis of Wuhan (www.who.int/csr/don/05-january-2020-pneumonia-of-unkown-cause-china/en/). One year later more than 100 million people in 191 countries have tested positive for SARS-CoV-2 and more than 2 million people have died from Coronavirus Disease 2019 (COVID 19) (https://coronavirus.jhu.edu/map.html).

In line with global data, most infections in Austria are found in the 50-64 age group, followed by the 35-49 age group [4, 8, 9]. In contrast to this, the majority of patients who died of or with COVID 19 can be found in the age group older than 80, with mortality rate of > 27.31%. With this data in mind and after the first partly dramatic reports of COVID 19 outbreaks in individual nursing home facilities in the USA [10-13], the effects of the COVID 19 pandemic on five nursing homes that cared for 889 residents in Austria were examined. The question to be answered was whether the measures taken by the responsible persons were appropriate to prevent an outbreak within the individual facilities. To detect even priorly unrecognized infections, the present study evaluated the prevalence of neutralizing antibodies against the SARS-CoV-2 virus in residents and employees in nursing homes. Prevalence of neutralizing antibodies in different population segments have been previously evaluated, but data on neutralizing antibodies prevalence particularly in nursing homes have been so far scarce [14-16].

After approval by the Ethics Committee of Caritas of the Archdiocese of Vienna and a written declaration of consent, the prevalence of neutralizing antibodies against the SARS-CoV-2 virus in the blood serum of residents and employees of 5 nursing homes of a private nursing home provider was analyzed. Administrative staff members of the private nursing home provider without personal contact to the facilities were chosen as control group. All included residents and staff members were interviewed in advance whether they had contact to a SARS-CoV-2 positive person and whether they had a proven infection with SARS-CoV-2 since January 2020. Furthermore, they were asked if they remembered any signs of a respiratory infection or fever within the first half year of 2020. Additionally, in the group of the residents the medical history was screened for COVID 19 specific and non-specific symptoms.

At the time of blood sampling, the result of at least one PCR of a nasopharyngeal swab was available for residents and employees of the nursing homes. Infections with SARS-CoV-2 were known in four of the five facilities. The blood samples were evaluated by the Austrian Agency for Health and Food Safety-Institute for Medical Microbiology and Hygiene. To rule out false positive and false negative results, a total of 4 different antibody tests were carried out on each blood sample. Two rapid lateral-flow antibody tests WANTAI SARS-CoV-2 AbRapid Test (Beijig Wantai Biological Pharmacy Enterprise Co., Ltd., Beijing, China) and TAmiRNA-SARS-CoV-2 (TAmiRNA GmbH, Vienna, Austria) were used according to manufacture information. Additionally, the LIAISON® SARS-CoV-2 S1/S2 IgG (DiaSorin S.p.A, Italy) a qualitative chemiluminescent immunoassay (CLIA) and the Wantai SARS-CoV-2 Ab ELISA (Beijig Wantai Biological Pharmacy Enterprise Co., Ltd., Beijing, China) detecting Antibodies against the Receptor-Binding-Domain were used and applied according to the manufacture’s manual. In all samples showing at least one positive result, a neutralization assay was done. In short, Vero 76 clone E6 cells (CCLV-RIE929, Friedrich-Loeffler-Institute, Riems, Germany) were cultured in minimum essential medium Eagle (E-MEM) with Hank's balanced salt solution (HBSS) (BioWhittaker, Lonza, Szabo Scandic, Austria) supplemented with 10% fetal bovine serum (Corning, Szabo Scandic, Austria) (FBS). Vero 76 clone E6 cells were used for the neutralization assay and to determine the virus fifty-percent tissue culture infectious dose (TCID50) according to Reed and Muench [17] Vero E6 TMPRSS-2 (provided by Stefan Pöhlmann; Deutsches Primatenzentrum, Göttingen, Germany) - initially described in Hoffmann *et al*. [18] - were cultured in Dulbecco’s modified Eagle’s medium (DMEM) with 10% fetal bovine serum (FBS) and were used for growing virus stocks. The virus used for the neutralization assay was originally isolated from a clinical specimen (nasopharyngeal swab), taken in mid-March 2020 from a 25-year-old male patient in Lower Austria and further passaged twice on Vero E6 TMPRSS-2 cells. The neutralization assay was set up in flat-bottom 96-well tissue culture plates. Human sera were heat-treated for 30 min at 56°C and diluted 1 to 4 in triplicates in serum-free DMEM medium as starting point for the assay. Two-fold serially diluted sera were incubated with an equal volume of 50 μl SARS-CoV-2 at a minimum of 2,000 tissue culture infectious dose 50% (TCID50)/ml) for 90 min at 37°C. Next, 25,000 Vero 76 clone E6 cells were added to the serum/virus mixture in each well in a volume of 100 µl in EMEM supplemented with 10% FBS and incubated for 4 days at 37°C, 5% CO_2_ in a humidified incubator. The CPE in every well was scored under an inverted optical microscope and the reciprocal of the highest serum dilution that protected more than 50% of cells from CPE was taken as the neutralizing [17, 18].

After the analysis of the blood samples, the results of the different tests were described and the prevalence of neutralizing antibodies in nursing homes was calculated with binominal confidence intervals In a second step, the prospectively collected data were connected to data from screening examinations and data from contact tracing These data were obtained as part of the measures to prevent the virus from spreading within the facilities In March 2020 the private operator of the nursing homes defined three independent core measures First of all, access to the long-term care facilities was granted exclusively to residents and employees All visitors, as well as external service providers (for example hairdressers, foot care or physiotherapists) were denied access to the living areas and residents were forbidden to walk outside the facility Secondly, a major focus was placed on the prevention of infections through correct behavior As part of the risk communication, all employees were regularly informed about general behavioral guidelines, the isolation and quarantine concepts, as well as the necessary protective equipment in the various areas Great attention was paid to correct hand hygiene, avoiding shaking hands, keeping people at least 1.5 meters apart (as far as possible) and wearing mouth and nose protection by employees and residents (if tolerated) for all social contacts that do not allow the required distance of >1.5m (eg, care) The third point focused on regular screening of residents and employees The body temperature of all residents was recorded at least once a day starting in the middle of March 2020 All residents were examined daily for new symptoms that could be associated with a COVID 19 disease Additionally, employees were screened by clinical examinations on a regular basis All employees who were in direct contact with residents were asked to independently monitor and document their health in order to protect the residents At the entrance area of the nursing homes, especially trained employees were checking the body temperature using an infrared thermometer Entering the facilities was only possible for symptom-free employees with a body temperature of <37.3 degrees In addition to the clinical screening, in the first half of 2020, at least once, a series of PCRs of nasopharyngeal swabs was performed at every facility on behalf of the health authority.

## Prevalence of neutralizing antibodies against SARS-CoV-2 in nursing home residents and implications for better protection

In total the blood serum of 1092 people was screened for the prevalence of antibodies against the SARS-CoV-2 virus. This included 465 residents of long-term nursing homes, 452 employees of these nursing homes and 175 persons from the administrative department.

**Table 1:** Number of positive results of a certain test, which could be found with the other tests.

	TAMI RNA Lateral (prior positive PCR)	WANTAI, Lateral Flow	Qualitative LIAISON®	ELISA, WANTAI
TAMI RNA Lateral Flow	46 (26)	28	32	38
WANTAI, Lateral Flow	28 (21)	29	23	25
qualitative LIAISON®	32 (21)	23	54	30
ELISA, WANTAI	38 (26)	25	30	54

In 92 persons at least one of the investigations carried out showed the presence of antibodies in the blood serum. The TAmiRNA-SARS-CoV-2 was positive in 46 persons, the WANTAI SARS-CoV-2 AbRapid Test was positive in 29 persons, the LIAISON® SARS-CoV-2 S1/S2 IgG was positive in 54 persons and the Wantai SARS-CoV-2 Ab ELISA was also positive in 54 persons. Only 21 persons showed a positive result in all four tests carried out. In 17 of these 21 (80.95%) persons, who showed a positive result in all four tests, an infection with the SARS-CoV-2 virus was identified in advance using a PCR of a nasopharyngeal swab. In contrast, only in 26 of the 92 persons (28.3%), who showed at least one positive result in one of the 4 antibody tests, a positive PCR identified an infection with the SARS-CoV-2 virus in advance. Table 1 shows how many positive results of a certain test, could be found in the other tests. The number of prior known positive PCRs in these persons is shown in brackets. 16 of these PCR tests were positive between April 19^th^, 2020 and May 13^th^, 2020, 7 were positive between March 26^th^, 2020 and April 19^th^, 2020, 3 were positive between May 13^th^, 2020 and June 6^th^, 2020 and one test was positive between June 6^th^, 2020 and June 30^th^,2020. In the group of the administrative staff there was no confirmed infection with SARS-CoV-2, but one person was put in quarantine by the health authorities without a test.

Of those 26 persons, who were tested positive prior the antibody tests, the TAmiRNA-SARS-CoV-2 and the Wantai SARS-CoV-2 Ab ELISA detected antibodies in all 26 persons and the WANTAI SARS-CoV-2 AbRapid Test and the LIAISON® SARS-CoV-2 S1/S2 IgG missed 5, but not same prior positive tested patients.

**Figure 1.**
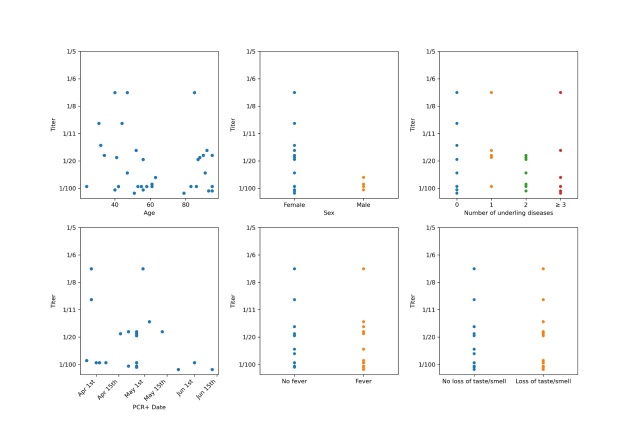
Correlation of the Titer of SARS-CoV-2 antibodies to age, time since infection, presence of fever or loss of taste. In total neutralizing antibodies against the SARS-CoV-2 virus were detected in 35 persons. The level of the antibody titer was in the range from 1:7 up to 1:32. Looking at the different titer levels one cannot extrapolate an obvious correlation of the level of the detected titer to age, time since infection, the presence of fever (Temp above 38 degree Celsius) or presence of a loss of taste.

As described above, if a positive result was found in one of the four performed antibody tests, the blood serum of these persons was examined for neutralizing antibodies against the SARS-CoV-2 virus. In total neutralizing antibodies as evidence of gone through infection with the SARS-CoV-2 virus were detected with different titer levels in 35 persons (17 residents and 18 employees). The range of the Titer was from 1:7 up to 1:320. Figure 1 shows, that one can extrapolate no obvious correlations of the level of the detected titer to sex, age, time since infection, the presence of fever (Temp above 38 degree Celsius), number of underling diseases or presence of a loss of taste.

**Figure 2.**
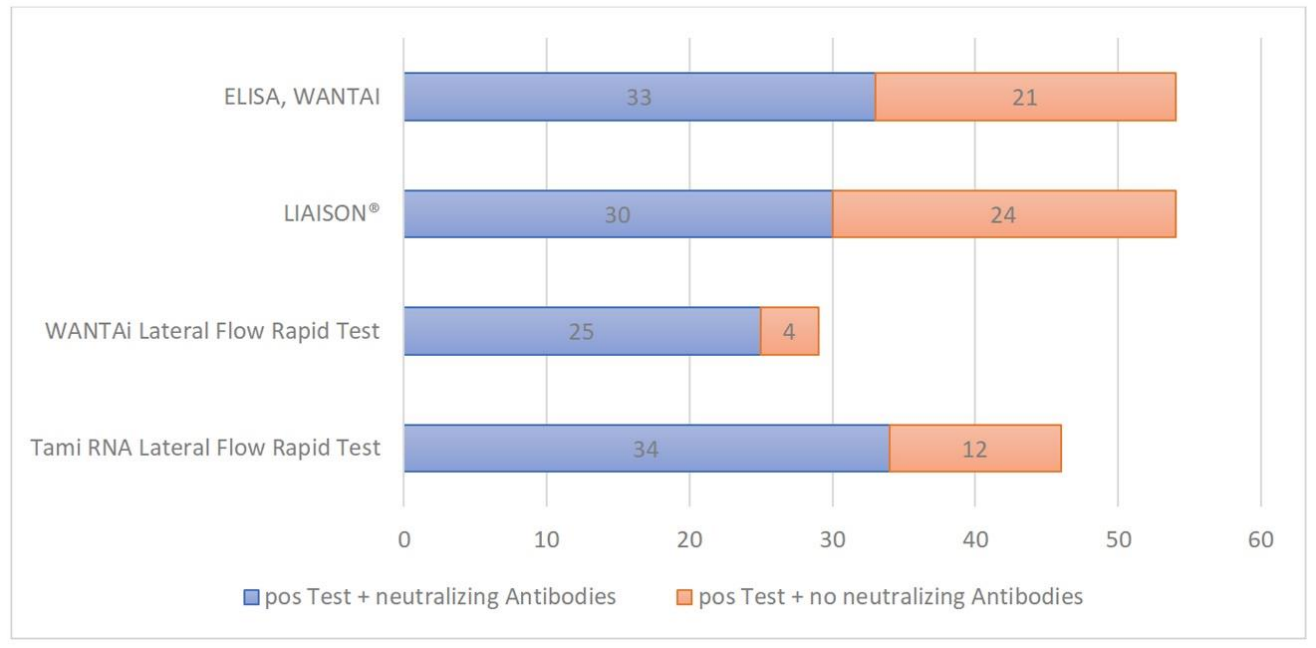
The presence of neutralizing antibodies in the blood serum of a person with a positive result in one of the previously performed antibody tests. In 92 blood samples (out of 1092 tested) at least one of the performed antibody tests was positive. In the subsequent screening for neutralizing antibodies against the SARS-CoV-2, neutralizing antibodies were found in a total of 35 of these 92 blood samples. The blue bar represents persons which showed a positive result in the test and as well showed neutralizing antibodies against the SARS-CoV-2. The orange bars symbolize people with positive test, where no neutralizing antibodies could be detected.

Figure 2 shows whether or not neutralizing antibodies were detected in the blood serum of a person with a positive result in one of the previously performed antibody tests. The blue bar represents persons which showed a positive result in the test and as well showed neutralizing antibodies. The orange bars symbolize people with positive test, where no neutralizing antibodies could be detected. In summary, the TAmiRNA-SARS-CoV-2 missed one of the persons with neutralizing antibodies against SARS-CoV-2, the WANTAI SARS-CoV-2 AbRapid Test missed 10, the LIAISON® SARS-CoV-2 S1/S2 IgG missed 5 and the Wantai SARS-CoV-2 Ab ELISA missed 2.

The retrospective analysis of all 5 included nursing homes showed that in 25 of the 35 persons (71.42%) with neutralizing antibodies against the SARS-CoV-2 virus an infection with the virus was proven in advance with a PCR of a nasopharynx swab. 4 of the persons were residents, and another 6 were employees in the nursing homes. Interestingly, for one resident who was tested positive in a PCR test in advance, no neutralizing antibodies could be detected. Nevertheless, this resident showed positive results in all other performed antibody tests. The 4 residents with neutralizing antibodies against SARS-CoV-2, who were never tested positive by a PCR of a nasopharyngeal swab were at least mobile in a wheelchair and had previous contact with a person who was tested positive to SARS-CoV-2. 2 of them lived in a multiple bedroom, one person lived together with 2 other persons tested positive with PCR in a four-bedroom. In total, from 1^st^ of January 2020 to the 15^th^ of July 2020, 90 infections with the SARS-CoV-2 virus were detected by regular clinical screening in combination with PCRs of a nasopharyngeal swab. An infection was detected in 66 residents and 24 employees from different professional groups. According to the contact tracing, it is very likely that the index patient was an employee in these four facilities. As part of a chain reaction, the virus was passed in the course of care activities on to residents and employees starting from the respective index-patient within the rooms and within the social rooms. At the present time, none of the affected employees, but 31 (46.97%) of the affected residents died with a proven infection with the SARS-CoV-2 virus.

Out of this data, one can extrapolate that compared to the administrative staff which showed a prevalence of neutralizing antibodies against the SARS COV 2 virus of 0.5% (CI 0.00-3.14%) in our sample, the prevalence of neutralizing antibodies in nursing homes in general is 3,7 % (CI 2.58 - 5.14). Whereas the prevalence in those facilities which have never been hit by an outbreak is 0, the prevalence in the facilities with an outbreak is up to 4.9% (CI 3.41-6.77%). Combining the retrospective data by adding the 31 persons who died with an infection proven by a PCR of a nasopharyngeal swab to the sample of neutralizing antibodies, the prevalence of a SARS-CoV-2 infection among residents and employees in nursing homes is 9.5% (CI 7.42-11.92%) when a facility is hit once. Furthermore, one can calculate that in nursing homes the attack rate in two-bedroom residencies is 66% and in the four-bedroom residencies up to 82%.

To the best of our knowledge, the present work is the second study that examines the prevalence of neutralizing antibodies against the SARS-CoV-2 virus within long-term care facilities. In November 2020 Ladhani et al demonstrated that five weeks after an initial SARS-CoV-2 outbreak 81.2% of surviving residents and 75.0% of staff showed neutralizing antibodies against the virus in six nursing homes in London [19]. These data are - as discussed in Ladhanis´ paper - in contrast to other cohorts including frontline health care workers [20-24]. But these data are also in contrast to the data from 5 long-term care facilities caring for more than 800 people in eastern Austria presented in this paper. The prevalence of neutralizing antibodies against the SARS-CoV-2 virus in those nursing homes in Austria, in which three independent core measures were initiated to reduce the risk of a transmission of SARS-CoV-2 was at the end of the first pandemic wave at a level of 3.7% in general. Whereas in the same region at the same time the prevalence in persons without personal contact to health care facilities was 0.5% and the prevalence in those facilities which have never been hit by an outbreak was 0, the prevalence in facilities with a documented outbreak was only up to 4.9%.

Patients in healthcare facilities have a right to be protected from preventable infections and of course resident’s safety is also of key importance to the facilities. At the time of the outbreak of COVID 19 diseases, those in charge of long-term care facilities moved in a virtually evidence-free room worldwide. To answer the question of whether the measures taken by the responsible persons were suitable to prevent an outbreak within the individual facilities, this study used four different antibody tests as an indirect method to detect a previous infection with SARS-CoV-2. In total, in 92 blood samples (out of 1092 tested) at least one of the performed antibody tests was positive. In the subsequent screening for neutralizing antibodies, neutralizing antibodies were found in a total of 35 of these 92 blood samples. As the TAmiRNA-SARS-CoV-2 and Wantai SARS-CoV-2 Ab ELISA in combination detected all 35 persons with neutralizing antibodies against SARS-CoV-2, a combination of these two tests might be a reliable prescreening strategy for further antibody studies. The limitation of the study is that for reasons of capacity in the laboratory only positive results were screened for neutralizing antibodies. But nevertheless, if one accepts the detection of neutralizing antibodies as the gold standard of the antibody tests, the present work demonstrated that all four antibody tests produced a huge number of false-positive results. Unlike tests that directly detect the antigen, where a false-positive result leads to the isolation of an actually uninfected person, false-positive results in antibody tests may simulate immunity and someone could also draw wrong conclusion from a positive result.

This study demonstrates that specific measures can prevent transmission within a health care facility. Nevertheless, the results also show that a risk reduction to 0% cannot be achieved. The prospective antibody test in conjunction with the retrospective analyzes showed that the SARS-CoV-2 virus was transmitted to 100 persons, 70 residents and 30 employees. While 92 of these infected persons were already known due to the screening together and contact tracing, 8 infections with the SARS-CoV-2 virus were missed by the taken measures. These 8 infections were confirmed in retrospect, as neutralizing antibodies against the SARS-CoV-2 virus could be proven in their blood serum. With a probability bordering on certainty these 8 persons were a danger to others within the respective facility at some point in the last 8 months. The moment a long-term care facility is hit by a SARS COV 2 infection, a chain reaction starts that can lead to up to nearly 50% fatalities among the affected residents [10, 12, 25]. If one adds the 31 persons who died with an infection proven by a PCR of a nasopharyngeal swab to the sample of the neutralizing antibodies, the prevalence of a SARS-CoV-2 infection among residents and employees in nursing homes is up to 9.5% (CI 7.42-11.92%). As risk is the product of the probability of the occurrence of damage and the damage to be expected in the event of the occurrence, one has to reduce the probability of virus transmission within nursing homes. Based on agent-based epidemiological model calculations Lasser et al. suggested that screening of the health care employees twice a week can reduce outbreak sizes even without a screening of residents [26]. In preparation for further pandemic waves, further studies are necessary to examine the effectiveness of different test strategies.

A worrying side finding of this study is, that in all 35 persons with neutralizing antibodies it was not possible to extrapolate any obvious correlations of the level of the detected titer to age, time since infection or the presence of symptoms like fever or loss of taste. Among those 25 people whose infection with the SARS-CoV-2 virus was previously documented by a PCR, one person had no neutralizing antibodies, 2 persons only had a titer of 1:7 and another person had a titer of 1:10. The person without any neutralizing antibodies was a resident 58 days after an infection with SARS-CoV-2 was proven with a PCR of a nasopharyngeal swab and at least 2 symptoms associated with COVID 19. One of the persons with the titer of 1:7 is a 40-year-old employee, the other person is an 85-year-old resident. The employee showed no symptoms in the course of the infection, the resident just as the 40-year-old employee with a titer of 1:10 showed typical symptoms of a COVID 19 disease. This finding indicates that 4 of 25 (14.8%) previously infected persons do not have sufficient humoral protection against a new infection 2-4 months after a COVID 19 disease.

The number of cases described is certainly too small to make general statements and future studies are needed about the development of herd immunity or the development of antibodies after vaccinations. This is the reason why we need to establish on one hand accurate and affordable test strategies, and on the other hand, disciplined adherence to the behavioral measures and intense clinical examinations will still be of great importance to protect the people we are caring for within high-risk populations. We need to realize that the SARS-CoV-2 virus has come to stay. The virus will not adapt, but society as a whole has to adapt in order to master this crisis successfully. We have to admit that we will not be able to detect 100% of all infections and neither herd immunity nor vaccination will most likely produce 100% protection. For this reason, as part of the preparation for further pandemic waves, solutions must be sought that guarantee the highest possible level of security and at the same time offer a balance between limited freedom and the best possible quality of life. To protect both the individual well-being and the well-being of society, it will be necessary not to demonize successfully established behavioral measures of the first wave, but to integrate them into everyday life.
